# Annotations

**Published:** 1895-11-02

**Authors:** 


### Annotations.

### Guy's, and Mr. Gladstone's Appeal.

The hearts of many people must have warmed towards Mr. Gladstone when they read his eloquent appeal in Wednesday's papers on behalf of Guy's Hospital. Mr. Gladstone went carefully into the question of expenses with the help of the "Hospital Annual," and made himself thoroughly acquainted with the financial needs and necessities of Guy's at the present time. The past week has been fruitful in appeals for personal service, and Mr. Gladstone's letter is a touching proof that everybody can do something effectual for others by giving themselves. At the present time the general and economical administration of Guy's Hospital is satisfactory, but we are afraid from what we hear that Mr. Gladstone's appeal must fail for the present. If Mr. Gladstone had first studied the constitution of Guy's, we believe his letter would have been withheld until certain modifications had been made in various directions. The present list of governors contains the names of some of the most respectable and best-known men in the City of London. It could be wished that they would devote a measure of personal service to Guy's Hospital at this juncture, and that they could be induced to attend the meeting of Governors on November 11th next, and to advocate the modifications in the conduct of their business which press for adoption. After a lengthened trial, it is admitted by most people with knowledge that the system of government by a treasurer is ill-suited to the requirements of a great hospital which needs the support of the public. The treasurer is apt to discourage the active co-operation of individual governors. Hence the attendance at committees, which are not too frequently held, gradually falls off; and such a system leaves the best interests of a great charity like Guy's too much in the hands of one man, who is less able to do the work thoroughly as years roll on. The present treasurer has given upwards of twenty years' service to the hospital, and has well earned the leisure which his increasing years entitle him to expect. We imagine that he would not offer any real objection to changes which would free him from responsibility, and place the management of Guy's to a much larger extent in the hands of the Court of Committees, which ought to meet every week, not once in six as at present, the head of each department being present when any business affecting him is dealt with. We should do but an ill-service to Guy's did we not say bluntly, that, unless these changes are brought about, Mr. Gladstone's appeal must largely fail, despite the great claims which Guy's undoubtedly has upon those able to give. Both the president and treasurer, after their long services, might properly be included in the new year's honours list. Their retirement would enable the present constitution to be modified in the directions indicated. If the governors will attend as they ought to attend the meeting on the 11th Nov., and express a wish there, as some have done privately, that these changes may be brought about, a small committee might be appointed to examine and report upon the present system of control, with a view to its modification. The difficulty now impeding the subscription of funds will then speedily disappear. We have often had the pleasure of being associated with Mr. Lushington in various works of importance, and we cordially recognise his invariable courtesy and the services he has rendered in various directions. Our suggestions are therefore made from fulness of knowledge and in a spirit of true friendliness to him and to Guy's.

### Abnormality or Madness.

Perhaps it is too much to hope that the wit of man will ever be able to devise a strict criterion which shall mark off sanity from madness. Yet the fog in which we are involved as to the extent to which man is responsible for what he does is becoming denser every day. It is a misfortune that this question has chiefly arisen and been discussed in regard to grave and serious cases—cases of murder, in which the alternative of insanity has been death, for it can hardly be denied that in many such cases the plea of insanity would hardly have been invoked, and certainly would not have been listened to, but for the knowledge that a bald verdict of guilty involved an irrevocable sentence. Two very recent cases have shown on what very slight evidence of insanity, other than that derived from the crime itself, it is possible to escape the extreme penalty; and it becomes a matter of much interest to inquire to what extent the doctrine of irresponsibility, which is urged in murder cases, is to be taken as applicable to the little crimes and peccadilloes met with in daily life. We all know that such a malady as kleptomania exists, yet how sceptical we are when a case occurs, and how the people grin with satisfaction when they find a magistrate refusing a listen to such a plea. Kleptomania, however, is none the less a fact, and if sudden impulse be admitted as an excuse at all, we may well question whether it has not been the real cause of many crimes which nevertheless have received the punishment due to wilful malice. It is, however, in matters of ordinary conduct rather than of crime that the question of responsibility becomes of interest in our social relations. Clearly, so far as oddity can be taken as explaining and excusing crime, it should be admissible also as proof of that insanity the presumption of which is accepted as the crime's excuse. The doctrine is growing up that to be abnormal is to be irresponsible. But what a field of abnormality there is around us! Surely these odd people are not all insane! We have amongst us those who believe in the supernatural, and profess that in their comings in and goings out they are surrounded by spirits, who influence them and guide them in their daily life. Are they mad, and if not, how shall that man be excused, who, urged on by spirit voices in his ear, is driven with uncontrollable impulse to slay his neighbour? What, also, about immorality and defiance of custom; how far may man or woman outrage the social rules prevalent at the moment without risking the asylum? Everyone laughs at "The Woman Who Did," and says she was cracked upon her one idea. But how about her imitators in the flesh? Are they also mad? Are Socialists mad, or is it only that a rich Socialist is crazy while a poor one is a clever fellow? Who, indeed, is mad—the idle tramp who wanders o'er the country the summer through, no doubt suffering many discomforts but still enjoying life, or the honest worker who slaves in a mill, and lives in a slum, and dies in the workhouse? Normal man is often but a mean fellow, and we must be careful how we accept mere abnormality of conduct as proof of irresponsibility.

